# Biolistic Transformation of *Haematococcus pluvialis* With Constructs Based on the Flanking Sequences of Its Endogenous Alpha Tubulin Gene

**DOI:** 10.3389/fmicb.2019.01749

**Published:** 2019-08-02

**Authors:** Guanhua Yuan, Xiaoying Xu, Wei Zhang, Wenlei Zhang, Yulin Cui, Song Qin, Tianzhong Liu

**Affiliations:** ^1^Key Laboratory of Biofuels, Qingdao Institute of Bioenergy and Bioprocess Technology, Chinese Academy of Sciences, Qingdao, China; ^2^Shandong Provincial Key Laboratory of Energy Genetics, Qingdao Institute of Bioenergy and Bioprocess Technology, Chinese Academy of Sciences, Qingdao, China; ^3^University of Chinese Academy of Sciences, Beijing, China; ^4^Yantai Marine Economic Research Institute, Yantai, China; ^5^Key Laboratory of Coastal Biology and Biological Resource Utilization, Yantai Institute of Coastal Zone Research, Chinese Academy of Sciences, Yantai, China

**Keywords:** alpha tubulin, biolistic transformation, flanking sequence, *Haematococcus pluvialis*, selection marker

## Abstract

*Haematococcus pluvialis* has high commercial value, yet it displays low development of genetic transformation systems. In this research, the endogenous 5′ and 3′ flanking sequences of the constitutive alpha tubulin (*tub*) gene were cloned along with its encoding region in *H. pluvialis*, in which some putative promoter elements and polyadenylation signals were identified, respectively. Three selection markers of tub/aadA, tub/hyr and tub/ble with three different antibiotic-resistance genes fused between the endogenous *tub* promoter (Ptub) and terminator (Ttub) were constructed and utilized for biolistic transformation of *H. pluvialis*. Stable resistant colonies with introduced *aadA* genes were obtained after bombardments of either *H. pluvialis* NIES144 or SCCAP K0084 with the tub/aadA cassette, the efficiency of which could reach up to 3 × 10^–5^ per μg DNA through an established manipulation flow. Two key details, including the utilization of culture with motile flagellates dominant and controlled incubation of them on membrane filters during bombardments, were disclosed firstly. In obtained transformants, efficient integration and transcription of the foreign tub/aadA fragments could be identified through genome PCR examination and qPCR analysis, nonetheless with random style instead of homologous crossover in the *H. pluvialis* genome. The presented selection marker and optimized transforming procedures in this report would strengthen the platform for genetic manipulation and modification of *H. pluvialis*.

## Introduction

Astaxanthin (3, 3′-dihydroxy-ß-carotene-4, 4′-dione) is the strongest antioxidant in nature and has been widely used as an additive in health care products and cosmetics (Han et al., 2013). *Haematococcus pluvialis* is the best producer of astaxanthin in nature. On astaxanthin content in biomass, it surpassed all of the other organisms, simultaneously with the 3S, 3′S isomer as its predominant isoforms, which is a preferable form as the feed additive (Boussiba, 2000). Currently, *H. pluvialis* cultivation has been commercialized and the edibility of its whole-cell biomass has been approved by the FDA (Panis and Carreon, 2016). It is also considered as a good biological producer for recombinant pharmaceutical proteins (Saei et al., 2012).

In *H. pluvialis*, continuous efforts have been made to improve its cellular growth and level of astaxanthin accumulation through traditional mutagenesis (Cheng et al., 2016); however, its immature platform for gene transformation and expression limited application in fields of metabolic engineering and synthetic biology. To date, only four research groups have reported stable genetic transformation of *H. pluvialis*. A mutated endogenous *pds* (phytoene desaturase) gene conferring resistance to norflurazon was successively used to transform *H. pluvialis* through biolistic bombardments by Steinbrenner and Sandmann (2006) and Sharon-Gojman et al. (2015). Chloroplast transformation of *H. pluvialis* was achieved biolistically by Gutierrez et al. (2012) with an *aadA* (an aminoglycoside-3′-adenylyltransferase) gene driven by an endogenous *rbcL* (large subunit of ribulose bisphosphate carboxylase) promoter. An agrobacterium-mediated method was once proposed to transform *H. pluvialis*, which was based on a *hptII* gene driven by a plant-general CaMV35S promoter (Kathiresan and Sarada, 2009; Kathiresan et al., 2009). This method was not repeated successfully by the other researchers (Sharon-Gojman et al., 2015), and similar failed attempts may have been encountered. So there must be some operational details in *H. pluvialis* transformation waiting to be disclosed and more efficient genetic toolboxes are still needed for the establishment of its efficient genetic engineering system.

Alpha tubulin (*tub*) is a principal component of microtubules, which are ubiquitous constituents of eukaryotic mitotic spindles, cytoskeleton, cilia, and flagella (Bandziulis and Rosenbaum, 1988). Its promoter and terminator have been widely utilized for the construction of efficient screening markers in different organisms. The coding sequence (CDS) of the *tub* gene in *H. pluvialis* was firstly cloned by Eom et al. (2006) and its constitutive expression was shown by transcript analysis. In this work, the endogenous 5′ and 3′ flanking sequences of the alpha tubulin gene were cloned from *H. pluvialis*, and some key transcriptional elements were identified in them. Moreover, three antibiotic-resistance genes were each independently fused between the obtained flanking sequences. The resulting fusion cassettes were used to transform *H. pluvialis* biolistically. The transforming efficiency, the influencing factors, the integration and transcription of the introduced foreign DNA fragment were investigated and discussed together.

## Materials and Methods

### *H. pluvialis* Strains

*Haematococcus pluvialis* NIES144 and SCCAP K0084 were purchased from the Microbial Culture Collection at the National Institute for Environmental Studies and the Scandinavian Culture Collection of Algae and Protozoa, respectively. Both strains were maintained in BG11 medium at 25°C with continuous illumination of about 25–30 μmol m^–2^ s^–1^.

### PCR Cloning and Analysis

The regular PCR was performed as follows: a 10 min initial denaturation step at 95°C, followed by 30 cycles of denaturation for 30 s at 95°C, annealing 30 s at 57°C, extension 1 min per kilo bp sequence length at 72°C, and a final 10 min extension step at 72°C. The TransTaq DNA polymerase High Fidelity (HIFI) kit (TransGen Biotech, Beijing, China) and 2× PCR BestaqTM MasterMix (Applied Biological Materials Inc., Vancouver, Canada) were utilized for DNA cloning and PCR analysis, respectively. The sequence information of the primers was provided in Supplementary Table S1. The pEASY-T1 Cloning Kit (TransGen Biotech, Beijing, China) was utilized for TA cloning of the target sequence.

### Isolation of Genomic DNA

Genomic DNA of *H. pluvialis* was isolated using the PlantZol reagent (TransGen Biotech, Beijing, China). The suspension with *H. pluvialis* cells mixed with the PlantZol reagents was firstly grounded with an electric grinder (Sangon Biotech, Shanghai, China) on ice and incubated at 55°C for 15 min. After extraction with the isometric solution of isopyknic phenol/chloroform/isoamyl alcohol (25:24:1, by vol), the genomic DNA was precipitated from the supernatant with isopropyl alcohol. The obtained genomic DNA precipitate was dissolved with distilled water after washing with 70% alcohol and stored at −20°C.

### RNA Extraction and cDNA Synthesis

Total RNA of *H. pluvialis* was extracted using an RNAiso Plus kit (TaKaRa Bio Inc., Kusatsu, Japan). Harvested *H. pluvialis* paste, previously frozen with liquid nitrogen, was ground in a mortar and then mixed with RNAiso Plus reagent. After 5 min incubation at room temperature and then extraction with chloroform, the RNA was precipitated with isopropyl alcohol and dissolved with RNase-free distilled water after washing with 80% alcohol. The first-strand cDNAs were synthesized using a PrimeScript TM II 1st Strand cDNA Synthesis Kit (TaKaRa Bio Inc., Kusatsu, Japan). The resulting cDNA sample was stored at −70°C.

### Genome Walking

In order to obtain the 3′ flanking sequences of the alpha-tubulin without any available information, a partially overlapping primer-based PCR was used for genome walking (Li et al., 2015), with a Genome Walking Kit (TaKaRa Bio Inc., Kusatsu, Japan). Three specific synthetic primers of Tubsp1, Tubsp2, and Tubsp3 were designed. Each primer had a relatively high annealing temperature. Three rounds of PCR were performed for each walking process using the product of the previous PCR as a template for the next PCR. Each PCR reaction mixture had 1 × LA PCR Buffer II (Mg^2+^ plus) containing 0.4 mM dNTP mixture, 2.5 U TaKaRa LA Taq, 0.2 μM of each primer and a certain amount of template. The first round of PCR had three annealing stages: stage 1 was five high-stringency (65°C) cycles; stage 2 was one low-stringency (25°C) cycle; and stage 3 was thirty high-stringency (65°C) cycles. The next two rounds of PCR had two annealing stages: stage 1 was 30 high-stringency (65°C) cycles and stage 2 was 15 low-stringency (44°C) cycles. After agarose gel electrophoresis and gel extraction, the fragments were cloned into pEASY-T1 vectors and sequenced.

### Construction of Transforming Plasmids

A fusion PCR method was performed to fuse the genes of *aadA* (from a plasmid pHpluS1 presented from Prof. Vitalia Henríquez), *hptII* (from the plasmid pCAMBIA1305) and *sh ble* (from the plasmid pPha-T1) with the *tub* promoter and terminator of *H. pluvialis* NIES144, respectively. Taking the construction of the tub/aadA cassette as an example, three target fragments of Ptub, aadA, and Ttub were obtained by regular PCR with the primer pairs of tub-f1/Ptub(aadA)-r1, aadA-f2/aadA-r2 and Ttub(aadA)-f1/tubr3, respectively. The three purified DNA fragments in equal mol amounts were mixed with the *pfu* DNA polymerase in a 50 μL PCR reaction system. This mixture underwent a PCR fusion procedure as follows: a 10 min initial denaturation step at 95°C followed by thirteen cycles of denaturation for 30 s at 95°C, annealing 30 s at 55°C, extension 2–3 min at 72°C, and a final 10 min extension step at 72°C. The final mixture was utilized as a template for PCR amplification of the target tub/aadA fragment with the primer pair of tub-f0/tub-r0. The resulting fusion fragments were cloned into the plasmid pEASY-T1. Three verified plasmids were named as pEASY-tub/aadA, pEASY-tub/hyr and pEASY-tub/ble. Their sequences were submitted to Genebank with the accession numbers of MH752993, MH752994, and MH752992, respectively.

### Transformation of *H. pluvialis*

For biolistic transformation, *H. pluvialis* cells were grown in BYA medium as described by Cheng et al. (2018). Cultivation times were chosen based on their growth and the percentage of flagellate cells. Cells were harvested by centrifugation and suspended in BG11 medium to a cell density of 2–3 × 10^8^ ml^–1^. A 100 μl aliquot of cell suspension was layered onto a 25 mm mix cellulose ester filter membrane, which was placed on a BG11 solid plate for bombardment. The bombardment was performed with a PDS-1000/He Biolistic Particle Delivery system (Bio-Rad, United States) as described by Steinbrenner and Sandmann (2006). Plates were bombarded from a distance of 6 cm using 1350 psi rupture disks. DNA coated particles were prepared by mixing 50 μl M 17 tungsten particle solution (60 mg/ml in H_2_O) with 5 μl of a DNA solution (>1 μg/μl), 50 μl of 2.5 M CaCl_2_, and 20 μl of 0.1 M spermidine base. This was followed by a 10 min incubation on ice and twice centrifugal washes with 70% and then 100% ethanol before final resuspension in about 50 μl ethanol. A total of 10 μl of prepared DNA-coated particle solution was layered on a microcarrier for one bombardment. After the bombardment, the cells were washed from the filter membrane into about 25 ml of BG11 liquid medium for the incubation at 25°C with continuous illumination at 10–15 μmol m^–2^ s^–1^. After 48 h regeneration, *H. pluvialis* cells were screened on solid plates of antibiotic-containing TAP media at 25°C with continuous illumination of about 25–30 μmol m^–2^ s^–1^. The antibiotic concentration was determined after a sensitivity test. For tub/aadA, tub/hyr, and tub/ble, optimal screening conditions were 200 μg ml^–1^ of spectinomycin, 10 μg ml^–1^ of hygromycin and 5 μg ml^–1^ zeocin, respectively.

### Determination of Cell Growth, Flagellate Content, and Cell Viability

The cell density, flagellate percentage and viability were determined by hemocytometer counting using an Olympus BX53 microscope. The number of flagellate cells was judged visually by its flagella and cell morphology. Phenosafranine staining was used to determine cell viability; cells that stained into red were counted as dead. All results were obtained from ≥200 cells in repeated samples. Cell viability was also determined through its growth on a solid plate. An aliquot with calculated 200 *H. pluvialis* cells was plated on a solid medium plate and the colony was counted after 2 weeks of cultivation.

### Quantitative Real-Time PCR (qPCR)

The qPCR was performed with a Roche LightCycler480 system. A TB Green Master Mix (TB Green^®^Premix Ex Taq^TM^II, TaKaRa, Japan) was utilized for the amplification of target PCR products. A two-step PCR was applied as follows: a 30 s initial denaturation step at 95°C, followed by 45 cycles of denaturation for 5 s at 95°C, and annealing and extension for 20 s at 60°C. Relative quantification analysis was fulfilled based on a ΔΔCt method (Livak and Schmittgen, 2001). The primer pairs of aadA2f/aadAqr and tubqf/tubqr were used for the quantification of the *aadA* and the *tub*, respectively.

### Sequence Analysis

Sequence alignments were performed with Clustal X. The putative cis–regulatory elements of the promoter were analyzed using the PlantCARE databases.^1^ The nucleotide sequences of the tubulin genes along with their flanking regions from *H. pluvialis* NIES 144 and SCCAP K0084 were deposited in the GenBank database.

## Results

### Clone of the Alpha Tubulin (*tub*) Gene and Its Flanking Sequences in *H. pluvialis*

**FIGURE 1:**
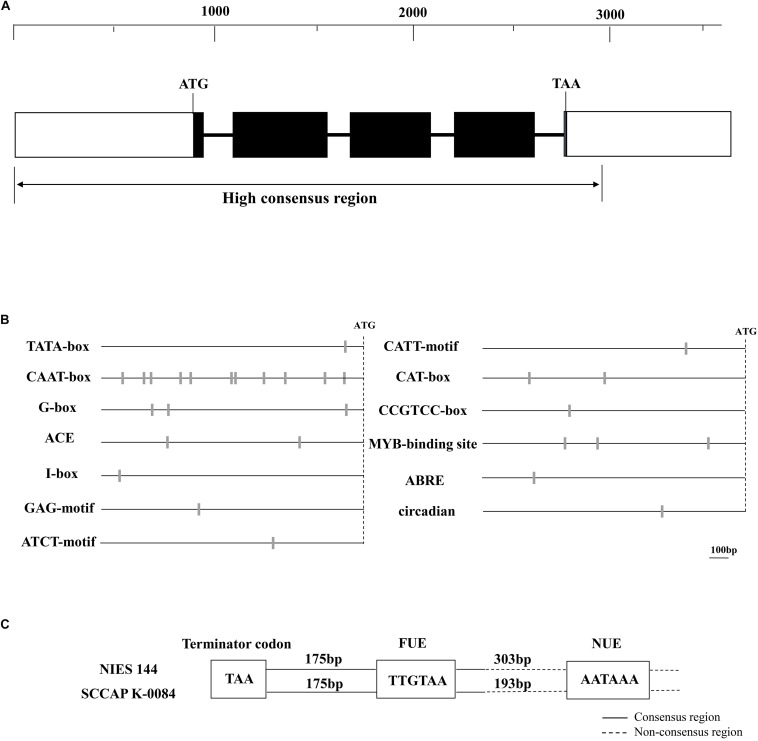
Maps of the cloned *tub* genes **(A)**, putative transcriptional elements in its 5′ **(B)** and 3′ **(C)** flanking regions of *Haematococcus pluvialis*. **(A)** The dark boxes, thicken lines and blank boxes represented the exons, introns and flanking regions of the *tub* gene, respectively. A ruler of DNA sequence lengths was provided above them. The double arrow line below indicates the region with high sequence consistency between *H. pluvialis* NIES144 and K0084. **(B)** The sites of predicted promoter elements are shown with gray bars on the dark lines, which indicate the 5′ flanking region of the *tub* gene. The site of the initiation codon (ATG) is shown with the dashed line. A DNA-length bar of 100 bp is provided. **(C)** In the 3′ flanking region of the *tub* gene, two putative polyadenylation signals with the motif sequences of TTGTAA and AATAAA were classified as the far-upstream element (FUE) and the near-upstream element (NUE), respectively. The dark and dash lines respectively indicated the consensus and non-consensus regions between *H. pluvialis* NIES144 and K0084. Their DNA lengths among them and the termination codon (TAA) are shown with text frames.

The *tub* gene and its flanking sequences were cloned and sequenced from both of *H. pluvialis* NIES144 and SCCAP K0084. Their sequences, with lengths of 3663 and 3430 bp, were obtained and deposited in the GenBank database under accession numbers MH752990 and MH752991, respectively. Figure 1A shows their common features. The *tub* gene consisted of five exons and four introns in *H. pluvialis*. Its encoding region had a length of 1356 nts only with a difference between the two strains at several nucleotide sites. The *tub* gene and its 5′ flanking region were cloned from PCRs with primer pairs of tubulin-F/tubulin-R and Ptub-h1/Ptub-h3, respectively, which were designed mainly based on the sequence data from accession numbers of AY894136.1 and GQ470501.1 of Genebank. Respectively, 909 and 908 bp of 5′ flanking sequences were obtained for *H. pluvialis* NIES144 and SCCAP K0084, which were identical except for two divergent nucleotide sites. The extended sequencing toward the 3′ flanking region of the *tub* gene was performed through a genome walking strategy. Respectively, 846 and 610 bp of 3′ flanking sequences were obtained for the two strains. Except for about 288 bp sequence after the termination codon, there was little sequence similarity in the rest of the 3′ flanking region.

### Putative Transcriptional Elements in the Flanking Regions of the *tub* Gene

Possible promoter elements and polyadenylation signals were predicted in the 5′ and 3′ flanking regions of the *tub* gene respectively, which are conservative in two *H. pluvialis* strains. As shown in Figure 1B, one TATA-box was detected at −78 bp from the ATG start codon and eleven putative CAAT-boxes were scattered from −836 to −134 bp in the 5′ flanking region. Both of them belong to the common cis–acting element, universally existing in eukaryotic promoters. Some regulatory promoter motifs were also found, such as elements involved with light response (G-box, ACE, CATT, ATCT, GAG and I-box), meristem expression (CAT-box) and specific activation (CCGTCC-box), abscisic response (ABRE), circadian control (circadian) and MYB binding sites recognizing MYB transcription factors. In the 3′ flanking region of the *tub* gene, two possible polyadenylation signals were identified as the results in Figure 1C. They have the conservative sequences of TTGTAA and AATAAA and are classified as the far-upstream element (FUE) and the near-upstream element (NUE), respectively. The TTGTAA motif was located at 175 bp downstream of the TAA termination codon in either of two *H. pluvialis* strains, and its adjacent sequences showed high consistency between them, while the AATAAA motif was located in the non-consensus region of the *tub* 3′flanking region. In its adjacent region, there was none of any similarity identified except for itself.

### Optimized Biolistic Transformation of *H. pluvialis*

**FIGURE 2:**
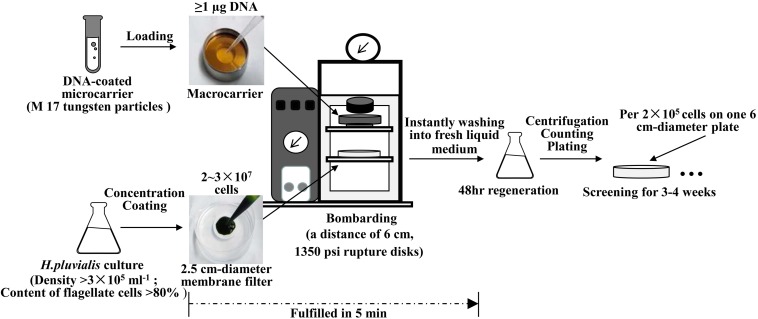
Flow diagram of biolistic transformation of *H. pluvialis*.

**FIGURE 3:**
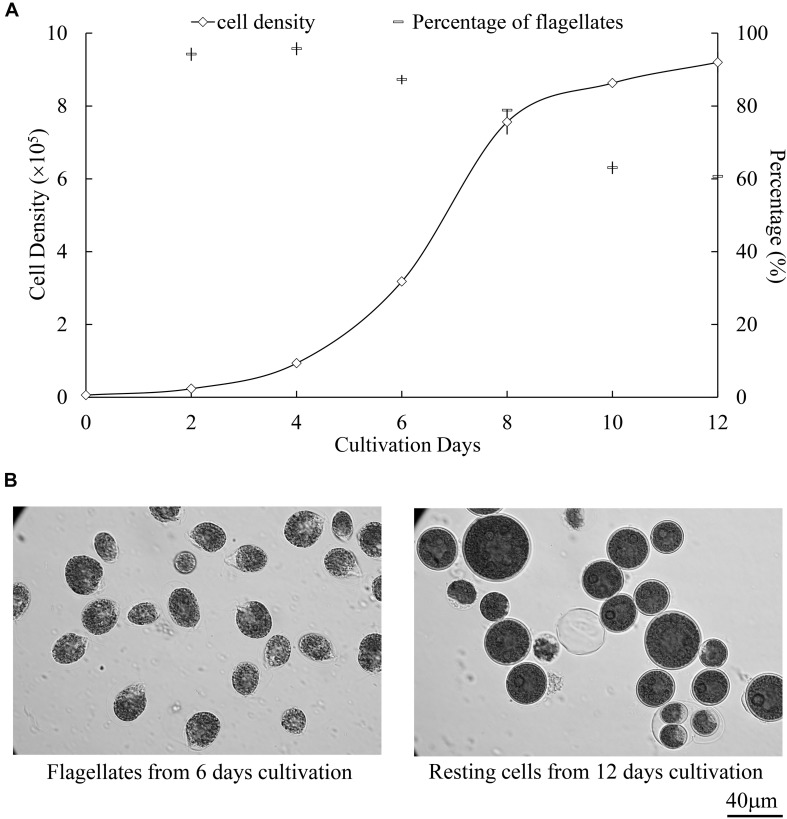
Growth and cellular differentiation at different cultivation stages of *H. pluvialis* NIES144. **(A)** Change of cell density and flagellate content in the culture of *H. pluvialis* NIES144. All the data are average ±SD from triplicate samples. **(B)** Microscopic photos of the motile flagellates and non-motile resting cells, respectively from 6- and 12-day cultivation of *H. pluvialis* NIES 144. A 40 μm scale bar is provided.

**FIGURE 4:**
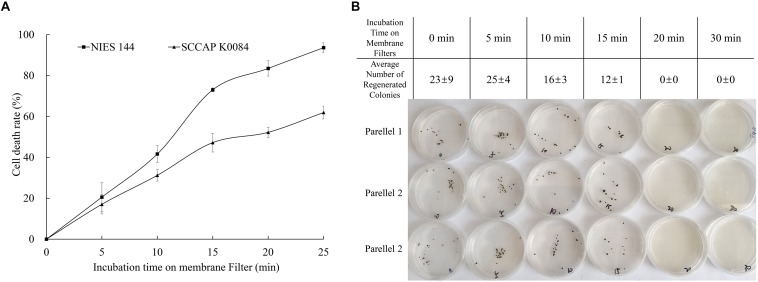
Changes of *H. pluvialis* viability with prolonged incubation on filter membranes. **(A)** Relative lethality obtained from a phenosafranine staining of *H. pluvialis* NIES144 and K0084 after different incubation time on filter membranes; **(B)** regeneration efficiency on solid medium plates after different incubation time on filter membranes of *H. pluvialis* NIES144. The data are average ±SD from three parallel plates.

As illustrated in Figure 2, optimized biolistic procedures were established for the transformation of *H. pluvialis*. It was suggested that M17 tungsten particles work as DNA carriers because gold particles were easy to adhere to the inner wall of eppendorf tubes, which might lead to difficulties in the DNA coating process. And the particles coated with at least 1 μg DNA were utilized for one bombardment. For the successful transformation of *H. pluvialis*, it is necessary to choose a logarithmic culture with vegetative flagellate cells in the majority. As shown in Figure 3, the motile flagellate tended to convert into the non-motile resting stage with extended cultivation. When a late-stage culture after 10 days of cultivation was used for bombardments, no successful transformation was once achieved. Therefore, a 6-day culture of *H. pluvialis* NIES144 was chosen, which had a cell density of 3.18 × 10^5^ ml^–1^ together with a flagellate content of 87.25%. Before bombardments, about 2–3 × 10^7^ of cells were condensed onto a 2.5 cm-diameter filter membrane, which could guarantee all of the cells in the center of bombarding range and gain full bombardments. It is very necessary to avoid long-time incubation of these bombarded cells on filter membrane, because *H. pluvialis* flagellates had bad survival after long-time incubation on it. The results of phenosafranine staining in Figure 4 show that the cellular vitality of *H. pluvialis* started to decrease after 5 min incubation on the membranes, and they could not be regenerated on medium plates after the incubation extended to 20 min or more. Thus, *H. pluvialis* cells after bombardments were washed immediately into fresh liquid media for 48 h regeneration, and the period of coating, bombarding and washing were always controlled within 5 min in this work. For the sake of stable screening and statistics of resistant colonies, per aliquot with about 2 × 10^5^ cells were spread on 1 screening plate with a 6 cm diameter for about 3–4 weeks of cultivation. It is very important for a successful transformation to use a double-layer plating instead of direct spreading.

### Application of Different Selection Markers Driven by Endogenous *tub* Flanking Sequences

**FIGURE 5:**
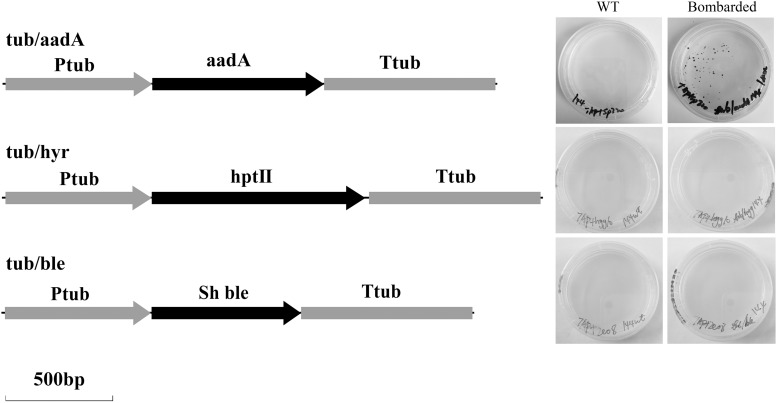
Maps of three selection cassettes of tub/aadA, tub/hyr and tub/ble, and screening results after bombardments of *H. pluvialis* NIES 144 with them. *H. pluvialis* cells were bombarded with the plasmids exhibiting tub/aadA, tub/hyr, and tub/ble, respectively. The obtained cells (Bombarded) were screened, respectively, at 200 μg spectinomycin ml^–1^, 10 μg hygromycin ml^–1^, and 5 μg zeocin ml^–1^ with the unbombarded cells as controls (WT). Ptub, the endogenous promoter from the 5′ flanking region of the *tub* gene; Ttub, the endogenous terminator from the 3′ flanking region of the *tub* gene; aadA, the spectinomycin-resistance gene from pHpluS1; hptII, the hygromycin resistance gene from pCAMBIA1305; sh ble, the zeocin resistance gene from pPha-T1.

**FIGURE 6:**
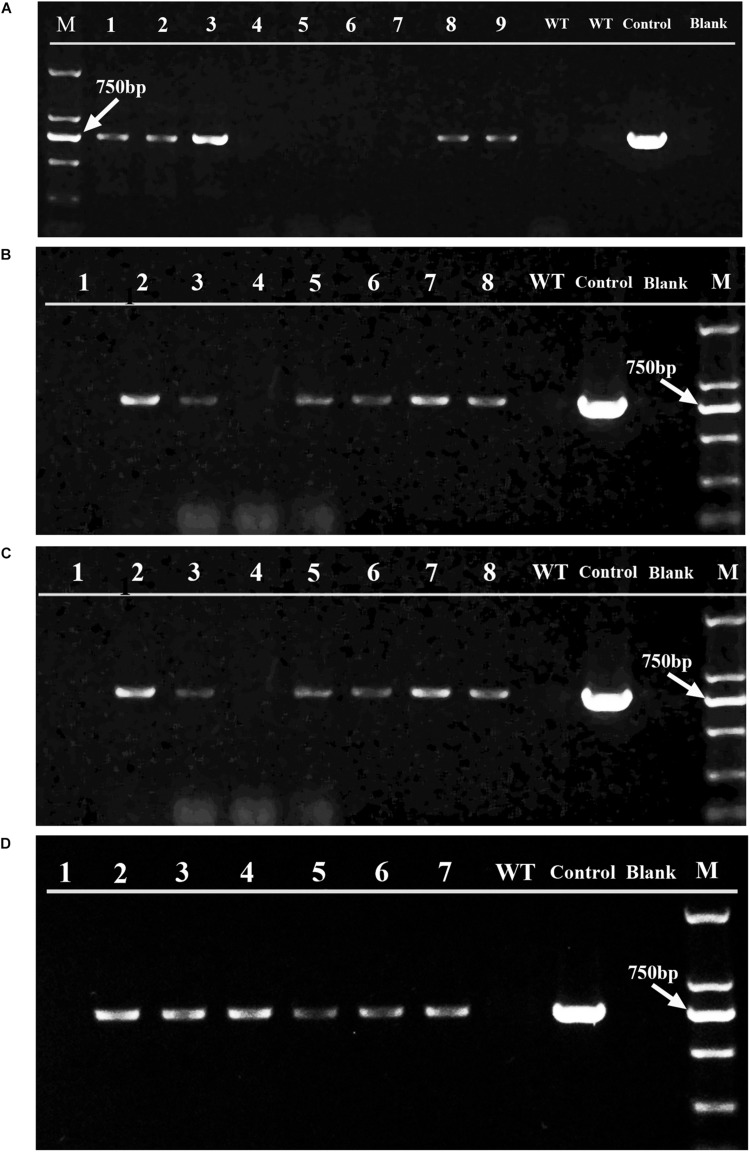
PCR examination of the *aadA* gene in the spectinomycin-resistant clones obtained from different bombardments. Panels **(A,B)** are the gel analysis results of resistant clones, respectively from *H. pluvialis* NIES 144 and K0084 bombarded with supercoiled pEASY-tub/aadA. Panels **(C,D)** were respectively from *H. pluvialis* NIES 144 and K0084 bombarded with linear PCR products of tub/aadA. The PCRs were performed with the primer pair aadA2f/aadA2r, which are specific to the *aadA* gene and have a target band of 744 bp in the positive transformants. The genomic DNAs of independent spectinomycin resistant clones obtained from these bombardments (Lane 1–9) were utilized as templates, with genomic DNAs of their wild type *H. pluvialis* (Lane WT), the plasmid DNA of pEASY-tub/aadA (Lane Control) and ddH_2_O (Lane Blank) as contrasts. A DL 2000 DNA marker (TAKARA) with six molecular weight bands (2000 bp, 1000 bp, 750 bp, 500 bp, 250 bp, 100 bp) (Lane M) were utilized and its 750 bp bands is indicated with a white arrow.

As shown in Figure 5, three selection markers with *aadA*, *hptII*, and *sh ble* genes fused between 5′ (Ptub) and 3′ (Ttub) flanking sequences of the endogenous *tub* gene were constructed and applied in *H. pluvialis* transformation, which encoded the spectinomycin, hygromycin and zeocin resistance genes, respectively. Stable spectinomycin-resistant colonies could be screened out after bombardments with the tub/aadA marker, in the form of either supercoiled plasmids or linear PCR fragments. Table 1 showed that the resistant-colony frequency could reach 0.4–3.0 × 10^–5^ per μg DNA. These resistant colonies were further checked through genome PCR analysis with the *aadA*-specific primer pair of aadA2f/aadA2r. As shown in Figure 6, the foreign *aadA* gene could be detected in most of these resistant clones of *H. pluvialis* NIES144 or SCCAP K0084 after bombardments with the supercoiled plasmid DNA or the tub/aadA fragment. To confirm the stability of the transgenic colony, these transformants were sustained in media with and without spectinomycin for at least three rounds of cultivation. For both strains, the foreign *aadA* gene was stably maintained through generations. For the markers of either tub/hyr or tub/ble, none of any positive transformants were obtained after repeatedly attempting biolistic transformation. Clones could form on the selection plates with gradually decreased antibiotic concentration, but it also led to the formation of false-positive ones due to incomplete inhibition of untransformed cells and none of any integration of introduced DNA fragments could be detected through genome PCR examination.

**TABLE 1:** Frequencies of resistant colonies achieved from different bombardments with the tub/aadA cassette.

**DNA**	**Strain**	**DNA quantity (μg)^a^**	**Average number of resistant colonies^b^**	**Total number of resistant colonies^b^**	**Colony frequency per μg DNA^c^**	**Colony frequency per DNA molecule^d^**
pEASY-tub/aadA	NIES 144	1.71	193.13 ± 22.43	1545	3.0 × 10^–5^	1.9 × 10^–16^
		1.18	58.75 ± 13.39	470	1.3 × 10^–5^	8.2 × 10^–17^
	K0084	1.71	181.00 ± 40.81	1405	2.7 × 10^–5^	1.7 × 10^–16^
		1.18	49.86 ± 37.42	431	1.2 × 10^–5^	7.6 × 10^–17^
Tub/aadA fragment	NIES 144	1.03	19.83 ± 9.75	119	0.4 × 10^–5^	9.4 × 10^–18^
	K0084	1.03	37.72 ± 22.52	285	0.9 × 10^–5^	2.1 × 10^–17^

*^*a*^Average DNA usage calculated from DNA concentration utilized in preparation of DNA-coated particles. ^*b*^About 3 × 10^7^ cells were bombarded for each experiment and screened on multiple solid plates. ^*c*^Total resistant colony numbers divided by cell numbers and DNA usage in one bombardment. ^*d*^Colony frequency per μg DNA multiplied by the DNA molecular weight and divided by Avogadro constant (6.02 × 10^–23^). The molecular weights (MW) of pEASY-tub/aadA and the Tub/aadA fragment were respectively 3806577 Dalton and 1416792 Dalton.*

### Integration of the Foreign DNA in the *H. pluvialis* Genome

**FIGURE 7:**
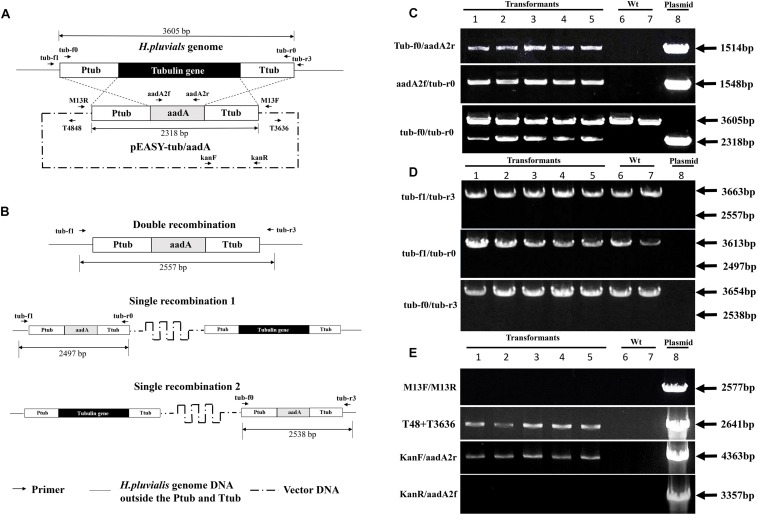
Illustration of potential homologous-recombinant regions **(A)**, possible homologous integration styles **(B)** of the plasmid pEASY-tub/aadA in the *H. pluvialis* genome, and genome PCR results for identification of the introduced tub/aadA cassette **(C)**, the homologous recombinant products **(D),** and the vector bone part **(E)** in five independent transformants of *H. pluvialis* NIES 144 bombarded with the plasmid pEASY-tub/aadA. The plasmid pEASY-tub/aadA has a DNA length of 6239 bp, on which the tub/aadA cassette exhibited an *aadA* gene driven by the endogenous *tub* promoter (Ptub) and terminator (Ttub) of *H. pluvialis*. Homologous crossovers of the introduced pEASY-tub/aadA with the genome of *H. pluvialis* at the regions of Ptub and Ttub (crossed dash lines) could produce three types of possible products, including one double recombination (substitution of the aadA gene with the *tub* gene) and two single recombinations (insertion of the pEASY-tub/aadA into the *H. pluvialis* genome). Integration of the tub/aadA cassette in *H. pluvialis* was confirmed by genome PCRs with three primer pairs of tub-f0/aadA2r, aadA2f/tub-r0, and tub-f0/tub-r0, which respectively had specific PCR bands of 1514, 1548, and 2318 bp in the positive transformants. Two primers of tub-f1 and tub-r3 were designed at the flanking regions of the Ptub and Ttub of the *H. pluvialis* genome. Their combined primer pairs of tub-f1/tub-r3, tub-f1/tub-r0, and tub-f0/tub-r3 were utilized for detection of the possible homologous recombinations. In their results, the absence of 2557, 2497, and 2538 bp indicated non-existence of the homologous recombination in the five transformants. In the results of tub-f0/tub-r0, tub-f1/tub-r3, tub-f1/tub-r0, and tub-f0/tub-r3, all PCR bands of 3605, 3663, 3613, and 3654 bp just targeted the wild-type *tub* gene in *H. pluvialis*. In the results of T48/T3636 and KanF/aadA2r, the bands of 2641 and 4363 bp in the transformants indicated the vector backbone of the plasmid pEASY-tub/aadA was integrated together with the tub/aadA cassette. Additionally, the absence of the 2577 and 3357 bp bands meant the plasmid did not exist with supercoiled forms in their genome. Sites of the primers are indicated with dark arrows on the maps. Target PCR bands were showed in lanes using templates of five independent transformants (1–5), the wild type *H. pluvialis* (6, 7) and the pEASY-tub/aadA DNA (8), indicated with dark arrows and text boxes of DNA length.

To investigate how the foreign DNA was integrated into the *H. pluvialis* genome, the obtained transgenic clones were examined by genome PCR analysis with different primer pairs. In Figure 7, the designed sites of the primers on the *H. pluvialis* genome or on the plasmid pEASY-tub/aadA were provided, and typical detection results for their target bands were given, which were obtained from five independent transgenic clones of *H. pluvialis* NIES144 bombarded with pEASY-tub/aadA. Specific bands could be obtained through PCRs with the primer pairs of both tub-f0/aadA2r and aadA2f/tub-r0, which proved that the tub/aadA cassette was completely integrated into the genome of the transformants. A 2641bp vector band was also identified in the five transgenic clones through PCRs with primer pair of T4848/T3636. It was proved by the 4363bp band obtained from the primer pairs of kanF/aadA2r that the vector part of the pEASY-tub/aadA was integrated together with the tub/aadA cassette into the genome of the five transgenic clones. None of any specific band could be obtained with the primer pairs of both M13F/M13R and kanR/aadA2f in them, which excluded the possibility that the plasmid existed in the transformants genomes with supercoiled forms. In the genomes of the transgenic clones, the *tub* gene (3605 bp) and the tub/aadA cassette (2318 bp) could be detected together using the primer pair of tub-f0/tub-r0. To detect possible homologous crossover in the transformants, two primers of tub-f1 and tub-r3, which were respectively located in the lateral region of Ptub and Ttub, were designed. As shown in Figure 7B, possible homologous double crossover and two types of single crossover were examined through PCRs with the primer pairs of tub-f1/Tub-r3, Tub-f1/tub-r0 and tub-f0/tub-r3, respectively. However, none of any bands indicative of homologous crossover was observed in them except for those wild-type bands of the *tub* genes in Figure 7D. Similar results were also obtained in other transformants of *H. pluvialis* NIES144 and SCCAP K0084 with either pEASY-tub/aadA or the tub/aadA cassette. This means that the foreign DNA was integrated into the *H. pluvialis* genome with non-homologous recombination.

### Quantitative Analysis of the *aadA* Gene in the Transformants

Quantitative real-time PCRs were performed to quantify the efficiency of genome integration and transcription of the introduced *aadA* genes in *H. pluvialis* transformants. Table 2 showed their quantitative results in the genomic DNAs and cDNAs obtained from five independent transgenic clones of *H. pluvialis* NIES144 bombarded with the pEASY-tub/aadA. The endogenous *tub* gene was utilized as a reference gene and the samples from wild-type *H. pluvialis* NIES144 as the control. Whether in the genomic DNA or the cDNAs, the five transformants had an obviously lower value of threshold cycles (Ct) for the *aadA* gene than did the wild-type samples and almost equal value for the reference *tub* gene. Normalized fold changes of the target gene (*aadA*) in these transformants to the wild-type cells were calculated with the *tub* gene as a balance based on a ΔΔCt method. There were calculated 174–396 times higher signals of *aadA* genes identified in the transformant genomes than the wild-type genome. And these fold changes were increased to 344–989 times in the cDNAs. This means that the *aadA* gene had been integrated into the genome and expressed efficiently in *H. pluvialis* transformants.

**TABLE 2:** Quantification of the *aadA* gene in five transformants of *H. pluvialis* NIES144 with pEASY-tub/aadA through a real-time PCR.

	Genomic DNA^*^			cDNA^*^		
		
	***Ct*^∗∗^**		**Fc^∗∗∗^**	***Ct*^∗∗^**		**Fc^∗∗∗^**
				
	**Balance(*tub*)**	**Target(*aadA*)**		**Balance(*tub*)**	**Target(*aadA*)**	
Wild type (Control)	16.57 ± 0.02	24.18 ± 0.43	\	17.70 ± 0.06	35.67 ± 1.52	\
Transformant1	16.36 ± 0.43	15.35 ± 0.49	396	16.2 ± 0.26	24.21 ± 1.82	989
Transformant2	16.42 ± 0.74	15.69 ± 0.34	326	16.05 ± 0.44	24.89 ± 1.41	555
Transformant3	16.31 ± 0.61	16.22 ± 0.54	209	15.78 ± 0.11	24.47 ± 1.49	620
Transformant4	16.14 ± 0.61	15.58 ± 0.12	289	16.49 ± 0.10	25.41 ± 0.47	526
Transformant5	15.39 ± 0.11	15.56 ± 0.05	174	16.49 ± 0.13	26.03 ± 1.31	344

^*^The template of genomic DNA or cDNA utilized in quantitative real-time PCR. ^∗∗^Threshold cycles: Lower *Ct* values indicate high amounts of template sequence. ^∗∗∗^Normalized fold change of the target gene (*aadA*) in transformants to the wild-type strain (control) with the *tub* as a balance gene based on a ΔΔ*Ct* method.

## Discussion

Active promoters and terminators are important constitutions of efficient transformation and expression vectors. It was generally thought that the utilization of those endogenous ones could greatly facilitate the integration and expression of foreign genes in the host genome (Radakovits et al., 2010). Researchers once chose those heterologous eukaryotic ones, such as SV40 (Teng et al., 2002) or CaMV35S promoters (Kathiresan et al., 2009), to drive foreign genes in *H. pluvialis*, but some only identified transient expression of reporter genes or some results with successful transformation could not be repeated widely (Sharon-Gojman et al., 2015). In this work, the complete sequence of the endogenous alpha tubulin (*tub*) gene, including its exons, introns, 5′ and 3′ flanking sequences were identified in *H. pluvialis*, which is a principal component of microtubules forming part of the cytoskeleton in eukaryotic cells and has a constitutive expression in *H. pluvialis*. There are several other genes with publicized records of their flanking sequences, including *bkt* (beta-carotene ketolase gene), *ipp1*(the isopentenyl pyrophosphate isomerase), *crtR-B*(carotene hydrolase gene) and *pds*, all of which were involved with astaxanthin synthesis in *H. pluvialis* (Meng et al., 2006; Steinbrenner and Sandmann, 2006; Wang et al., 2012). Until now, sequence acquisition of endogenous flanking regions was mainly based on regular genome walking or screening of genomic libraries due to the unreported or unfinished nuclear genome. This situation limits the quick access to and application of active promoters or terminators in the *H. pluvialis* nuclear genome.

Sequence analysis was performed on the flanking region of the *tub* gene for prediction of its potential cis–acting elements involved with transcription initiation, regulation or termination. In the *tub* 5′ flanking sequence, a TATA-box and eleven CAAT boxes were detected. The TATA box is located at the core promoter region and involved with accurate transcription initiation. In *Chlamydomonas* HSP70A promoter, its deletion decreased the transcription activity greatly (Lodha et al., 2008). The CAAT box commonly exists in many eukaryotic promoters and acts as an enhancer region. In nopaline synthase (*nos*) promoter, CAAT boxes are required for gene transcription in sufficient quantities (Dai et al., 1999). One TATA box and six CAAT boxes were identified in the *bkt* promoter of *H. pluvialis*, which showed universal promoter activity and could drive expression of antibiotic resistance genes in *Chlamydomonas reinhardtii* CC-849 (Wang et al., 2012). There were some regulatory motifs identified in the *tub* promoter, such as elements involved with light response (G-box, ACE, CATT, ATCT, GAG and I-box), meristem expression (CAT-box) and specific activation (CCGTCC-box), ABRE, etc. One ABRE motif showed an important regulation on expression of the carotenoid hydrolase gene of *H. pluvialis* (Meng et al., 2006).

Two possible poly(A) signals with the sequences of AATAAA and TTGTAA were identified in the 3′ flanking regions of the *H. pluvialis tub* genes, the corresponding RNA sequences of which were respectively recognized as the typical motifs in the NUE and the FUE (Hunt, 2008). The AAUAAA, the most well-known poly(A) signal, was firstly recognized in mammalian mRNAs within 30 nts upstream of the polyadenylation site (Proudfoot, 2011). In plants, it was classified into the NUE, which is an A-rich region with 6–10 nts length situated at 10–30 nts upstream from a polyadenylation site. Farther upstream of the poly(A) cleavage site (as far as 90 nts), a UUGUAA motif was once identified as the FUE with an important function in plant RNA polyadenylation (Sanfacon et al., 1991). Sequence analysis in *C. reinhardtii* showed that the UGUAA motif is predominant in the NUE (−30 to −10) instead of the FUE (Shen et al., 2008). Among previously publicized flanking sequences of *H. pluvialis* genes, few covered the 3′ flanking region except for the *pds* gene, while both of these two motifs were not identified in the 336-nts 3′ flanking sequence. This means that there should be other functional poly(A) signals to be identified.

Biolistic transformation is a universal genetic transformation technology (Elghabi et al., 2011) which has been used to target the genomes of both the nucleus (Steinbrenner and Sandmann, 2006) or the chloroplasts (Gutierrez et al., 2012) in *H. pluvialis*. Meanwhile, it was found that two undisclosed manipulation details involved with preparation and bombardment of *H. pluvialis* cells, respectively, influenced the success rate of its transformation. Firstly, it was important to choose a logarithmic culture with a high percentage of flagellate cells for bombardments. Motile flagellates and non-motile resting cells are two main cell types in the *H. pluvialis* culture (Gu et al., 2013). When a late-stage culture with non-motile resting cells is predominant was used for bombardments, no successful transformation was achieved. Teng et al. (2002) also reported that transient expression of the *lacZ* gene could be observed in motile cells but not in non-motile cells after biolistic bombardments. Meanwhile, cell differentiation of *H. pluvialis* could be influenced by many environmental factors, and there are also great differences among different *Haematococcus* species or strains (Borowitzka et al., 1991). Therefore, enriched flagellate cells of *H. pluvialis* were prepared in a BYA medium as previously reported by Cheng et al. (2018), and the culture phase used for bombardments was decided by its growth and flagellate content. Secondly, it is very necessary to avoid long-time incubation of *H. pluvialis* cells on filter membranes. Before bombardment, *H. pluvialis* cells need to be concentrated and coated onto a cellulose filter membrane. However, bad survival of flagellates was found after extended incubation (Figure 5). So the bombarded cells must be washed immediately from the membrane into a fresh liquid medium for regeneration, and the period of coating, bombarding and washing was always controlled in 5 min in this work. Neither of these two details were reported in previous research.

The selectable marker system is key to screening for transformants with stable integration of foreign genes into the *H. pluvialis* genome. Three different selection cassettes were used to transform *H. pluvialis* biolistically, which respectively have the *aadA*, *sh ble*, and *hptII* genes driven by newly cloned endogenous *tub* promoter and terminator. Stable transformants with integrated sequences and showing expression of *aadA* gene were obtained; however, constructs tub/hyr and tub/ble failed to produce transformants after multiple attempts. Kathiresan et al. (2009) once reported the successful transformation of *H. pluvialis* with the *hptII* gene driven by a CaMV35S promoter through an agrobacterium-mediated method. However, our previous attempts of *H. pluvialis* transformation with the plasmid pCAMBIA1305 exhibiting the same selection box failed through whether biolistic or agrobacterium-mediated methods in at least three different strains. The same unsuccessful experience was also reported by Sharon-Gojman et al. (2015), which was ascribed to the difference among species. The *sh ble* gene has been successfully utilized in the transformation of *C. reinhardtii* (Fischer and Rochaix, 2001), *Phaeodactylum tricornutum* (Zaslavskaia et al., 2000), and *Nannochloropsis oculata* (Li et al., 2014). The *sh ble* cassette driven by the PsaD promoter from *C. reinhardtii* was once integrated into the genome of *H. pluvialis* mediated by a mutated *pds* gene, which showed transcriptional expression but no screening activity (Sharon-Gojman et al., 2015). It was thought that unsuitable codon usage in the gene resulted in an insufficient protein expression in transformed cells, which should lead to the failed application of these antibiotic resistance genes.

The resistant colony frequency of 0.4–3 × 10^–5^ per μg DNA could be achieved on the transformation of either *H. pluvialis* NIES144 or SCCAP K0084, similar with previously reported efficiencies of 1.6 × 10^–5^ and 2 × 10^–5^ per μg DNA for the plasmids of pHpluS1 (Gutierrez et al., 2012) and pBS–pds (Sharon-Gojman et al., 2015). The PCR-amplified DNA fragments of the tub/aadA cassette were also utilized for the biolistic transformation of *H. pluvialis*. It began with an idea that the tub/aadA fragments should facilitate transformation due to the removal of the unnecessary vector part DNA, because it has more molecular numbers bombarded than the plasmid DNA under the same μg DNA. However, a relatively lower colony frequency per DNA molecule was obtained with the short DNA fragment than with the supercoiled plasmid DNA in either *H. pluvialis* NIES 144 or SCCAP K0084, as shown in Table 1. Similar results with short DNA fragments have also been reported in *P. tricornutum* (Kira et al., 2016). It was also reported in the electroporation of *Nannochloropsis* sp. that the PCR product showed much higher transformation efficiency than the linearized plasmid (Li et al., 2014). Therefore, it was inferred that the short PCR product may have a lower ligating efficiency with the DNA carriers, and that status would not exist in the electroporation method.

It was identified through genome PCR examination that the tub/aadA cassette was stably introduced into *H. pluvialis*, and the qPCR analysis proved that it was integrated into the genome and transcribed efficiently. Moreover, none of any homologous recombination events was detected in those obtained transformants through the examination of their characteristic PCR products. In eukaryotic microalgae, insertion of foreign DNA occurs principally at non-homologous or random sites, rather than homologous ones in the nuclear genome (Nelson and Lefebvre, 1995). It could be accompanied by the deletion of functional genes and lead to suicidal effects. As shown in Figure 7B, the double crossover at the regions of Ptub and Ttub would result in the deletion of the *tub* gene through substitution of its coding region with the *aadA* cassette. Because the *tub* gene may play an important role in cell growth and propagation in *H. pluvialis*, the double-recombinant clones may not survive during the transformant-screening process. Meanwhile either of the single crossovers kept the complete *tub* transcription frame, which should not be suppressed by the insertion of the plasmid pEASY-tub/aadA. Of course, there is a possibility that the insertion may influence the normal expression of those genes located upstream or downstream of the *tub* gene in the *H. pluvialis* genome. The homologous recombination was an important tool for gene-targeted knock-out (Kilian et al., 2011). However, it had limited success even in a model eukaryotic microalgae *C. reinhardtii* due to its low probability (Plecenikova et al., 2013). Currently, this method has been gradually substituted by those gene-editing methods such as zinc finger nucleases (ZFNs), transcription-activator-like effector nucleases (TALENs), and CRISPR-Cas9 ribonucleoproteins (Shin et al., 2016).

In conclusion, the stable nuclear transformation could be achieved in *H. pluvialis* NIES144 and SCCAP K0084 through biolistic bombardments, using the *aadA* gene driven by transcriptional elements flanking the endogenous *tub* gene. This approach enriches the molecular tools for genetic modification of *H. pluvialis*. Additionally, a biolistic transformation flow was well established, and two key cellular factors contributing to the success of *H. pluvialis* transformation were identified firstly in this research, which includes the importance of a high flagellate percentage in the culture and controlled incubating time of *H. pluvialis* cells on the filter membrane. Establishment of a stable and efficient transforming technology should facilitate research in metabolic engineering and synthetic biology in *H. pluvialis*.

## Data Availability

The raw data supporting the conclusions of this manuscript will be made available by the authors, without undue reservation, to any qualified researcher.

## Author Contributions

WeiZ, SQ, and TL contributed to the conception and design of the study. GY and XX carried out the basic molecular experiments including the DNA clone, plasmid construction, and phenotype analysis. GY and WenZ carried out the biolistic bombardment manipulation. GY, WeiZ, and YC performed the analysis work including the data treatment, sequence alignment etc. WeiZ wrote the first draft of the manuscript. GY, XX, and WenZ contributed to the Materials and Methods sections. YC, SQ, and TL took part in modifying the section of Discussion. All authors contributed to the manuscript revision, read, and approved the submitted version of the manuscript.

## Conflict of Interest Statement

The authors declare that the research was conducted in the absence of any commercial or financial relationships that could be construed as a potential conflict of interest.

## Footnotes

1 http://bioinformatics.psb.ugent.be/webtools/plantcare/html/
