# Supplementary material for: Biolistic Transformation of Haematococcus pluvialis With Constructs Based on the Flanking Sequences of Its Endogenous Alpha Tubulin Gene
Source: Front Microbiol. 2019 Aug 2;10:1749. doi: 10.3389/fmicb.2019.01749 (PMC6687776; doi:10.3389/fmicb.2019.01749)
Supplement: Supplementary file 1 [file Table_1.DOCX]

**Supplementary Table 1. The sequence information of the primers**

| Primer | Sequence |
| --- | --- |
| Tubulin-F | ACAGCACCCTTAAAGATGC |
| Tubulin-R | CGTGCGTCTCAGGCTTATTTG |
| Ptub-h1 | TGTATGCTGTCATGGCGC |
| Ptub-h3 | CGATCTTTAAGGGTGCTGT |
| Tubsp1 | GTACTAAACATGACGCTCAGGC |
| Tubsp2 | TATCCTAGCCTGCAGTGAAGCTG |
| Tubsp3 | TGCTGGTGCTGAATCGCTG |
| Ptub(aadA)-r1 | atcaccgcttccctcatCTTTAAGGGTGCTGTTTAAAGAG |
| aadA-f2 | ctctttaaacagcacccttaaagATGAGGGAAGCGGTGATCG |
| aadA-r2 | tgtgggcctgagcgtcatgtTTATTTGCCGACTACCTTGGTG |
| aadAqR | CAATGGTGACTTCTACAGCGC |
| Ttub(aadA)-f1 | ccaaggtagtcggcaaataaACATGACGCTCAGGCCCACA |
| Ptub(hyg)-r1 | caggctttttcatatctcatCTTTAAGGGTGCTGTTTAAACTGCG |
| hyg-f2 | cgcagtttaaacagcacccttaaagATGAGATATGAAAAAGCCTG |
| hyg-r2 | ctgtgggcctgagcgtcatgtCTACTCTATTTCTTTGCCCTCGG |
| Ttub(hyg)-f1 | ccgagggcaaagaaatagagtagACATGACGCTCAGGCCCACAG |
| Ptub(ble)-r1 | gaacggcactggtcaacttggccatCTTTAAGGGTGCTGTTTAAAC |
| ble-f2 | gtttaaacagcacccttaaagATGGCCAAGTTGACCAGTGCCGTTC |
| ble-r2 | ctgtgggcctgagcgtcatgTTCAGTCCTGCTCCTCGGCCAC |
| Ttub(ble)-f1 | gtggccgaggagcaggactgaACATGACGCTCAGGCCCACAG |
| tub-f1 | TGTATGCTGTCATGGCGC |
| tub-r3 | GGTTCATCTGCTGACGAGTGCT |
| tub-f0 | TCATGGCGCGACGCTGT |
| tub-r0 | CTTCAATAGTGCATTGCCAG |
| addA2F | CAGAGGTAGTTGGCGTCATCG |
| addA2R | CGACTACCTTGGTGATCTCGC |
| tubf | CTACCCGCGATGATTCGTTTG |
| M13F | GTAAAACGACGGCCAGT |
| M13R | CAGGAAACAGCTATGAC |
| KanF | GACTGGGCACAACAGACAAT |
| KanR | TACCGTAAAGCACGAGGAA |
| T48 | CCTGTCGTGCCAGCTGCATTA |
| T3636 | CAAGATGGATTGCACGCAGGTTC |
| tubqf | CAACGCAGTGTTTGAGCCT |
| tubqr | CAACGGCAGCATTCACATCC |
